# Evaluation of a 4-day repeated-dose micronucleus test in rat glandular stomach and colon using aneugens and non-genotoxic non-carcinogens

**DOI:** 10.1186/s41021-022-00241-6

**Published:** 2022-04-11

**Authors:** Emiko Okada, Yohei Fujiishi, Kazunori Narumi, Wakako Ohyama

**Affiliations:** Yakult Central Institute, Yakult Honsha Co., Ltd., 5-11 Izumi, Kunitachi-shi, Tokyo, 186-8650 Japan

**Keywords:** Micronucleus test, Gastrointestinal tract, Glandular stomach, Colon, 4-day treatment regimen, Aneugen

## Abstract

**Background:**

We previously developed a rodent gastrointestinal (GI) tract micronucleus (MN) test using the glandular stomach and/or colon, and evaluated this test method using several genotoxic carcinogens (clastogens) and genotoxic non-carcinogens; we demonstrated that this test method could detect genotoxic stomach and/or colon carcinogens with target organ specificity. In the present study, we further evaluated the sensitivity and specificity of the MN test for the rat glandular stomach and colon using three aneugens (colchicine, vinblastine sulfate, and docetaxel hydrate) and two non-genotoxic non-carcinogens (sodium chloride and sucrose).

**Results:**

Male Crl:CD (SD) rats were administered test compounds through clinical administration route (orally or intravenously) for four consecutive days and then examined for the micronucleated cell frequencies in the glandular stomach and colon. We observed that all three aneugens significantly and dose-dependently increased the micronucleated cell frequencies in the stomach and colon. In contrast, neither of the two non-genotoxic non-carcinogens increased the micronucleated cell frequency in these tissues. Notably, an increase in cell proliferation was observed in the glandular stomach of rats administered a stomach toxicant, sodium chloride, but this increase did not affect the induction of micronuclei in the gastric cells.

**Conclusions:**

In the present study, it was demonstrated that the glandular stomach and colon MN tests could detect aneugens as positive and could adequately evaluate non-genotoxic non-carcinogens as negative, including a chemical that enhances cell proliferation. These results provide important evidence supporting good performance of the rat glandular stomach and colon MN tests with a 4-day treatment regimen.

## Introduction

The erythrocyte micronucleus (MN) test using rodent bone marrow/peripheral blood is one of the standard genotoxicity tests in regulatory use and is valuable for evaluating the clastogenicity and aneugenicity of chemicals in vivo [1–3]. However, when a test chemical showing a positive result in an in vitro genotoxicity test gives a negative result in the erythrocyte MN test, it is necessary to conduct follow-up evaluation with a second in vivo test using appropriate target tissues and/or endpoints based on factors such as exposure, metabolism, and distribution in the drug development [1]. Regarding evaluation of orally administered substances, the gastrointestinal (GI) tract is useful being a direct contact site for the substances. In particular, the stomach is considered to be important because of its high exposure to orally administered substances. The colon is also useful for evaluating the genotoxicity of substances that reach the intestine via intestinal contents and/or bloodstream as well as substances that are metabolized by intestinal microbiota. For genotoxicity tests targeting the GI tract, the comet assay to detect DNA damage and the transgenic rodent gene mutation assay to detect gene mutations have already been established and listed in the OECD Test Guidelines [4, 5]; however, the GI tract MN test to detect chromosome damage has not yet been standardized for regulatory use.

Micronuclei are formed when cells with aberrations of chromosomes and/or mitotic apparatus divide into two daughter cells. The GI tract epithelium that has high cell proliferative activity is a suitable tissue for analysis of micronucleated (MNed) cells. GI tract MN tests using mice or rats have been developed by several researchers [6–13]. They have shown that the GI tract MN test could detect genotoxic stomach and/or colon carcinogens with target organ specificity and that the MNed cells in the stomach and colon increased relatively early after the administration of genotoxic agents (mainly clastogens). In our previous study using rats, the maximum frequency of MNed cells was observed at 48–72 h (glandular stomach) and 96 h (colon) after a single oral administration of genotoxic-carcinogens [13]. These time-response patterns of MN induction were considered to be ascribed to the relatively short turnover time (approximately 2–4 days) of the GI tract epithelium [14].

Based on such time-response patterns of MN induction, we developed a rat GI tract MN test combined with the bone marrow MN test with a 4-day treatment regimen [15]. The advantage of this regimen is that MN induction in cells of the glandular stomach, colon, and bone marrow can be simultaneously evaluated in the same rat. The test showed good performance using seven genotoxic carcinogens (clastogens) and two genotoxic non-carcinogens, and the results of the GI tract MN test correlated well with their carcinogenicity to the stomach and/or colon. Notably, *N*-methyl-*N′*-nitro-*N*-nitrosoguanidine (MNNG) and *N*-methyl-*N*-nitrosourethane (NMUT), genotoxic-stomach-carcinogens, which are known to be rapidly degraded by contact with high concentrations of thiols in the gastric mucosa after oral administration [16–18], were positive only in the glandular stomach, but not in the colon and bone marrow. As part of a collaborative study of the repeated-dose (RD) liver MN test in the Mammalian Mutagenicity Study (MMS) group, a subgroup of the Japanese Environmental Mutagen and Genome Society (JEMS), a collaborative trial of the GI tract MN test among six laboratories was conducted. After the technical transfer of the method, inter-laboratory reproducibility was confirmed using negative- and positive-control substances with a 4-day treatment regimen [19]. Furthermore, the glandular stomach and colon MN tests showed good performance even with the long-term RD regimen of 14 and/or 28 days [19, 20]. This evidence suggests that the GI tract MN test could be incorporated into a 28-day RD toxicity test, similar to the erythrocyte MN test. Based on these findings, the GI tract MN test was concluded to be a promising method to examine the clastogenicity of test chemicals in the stomach and/or colon of rats in the 6th and 7th International Workshop on Genotoxicity Testing (IWGT) [21, 22]. However, it was also mentioned that further data would be needed to identify the sensitivity and specificity of this test, in particular, validation of sensitivity using aneugens and specificity using both genotoxic and non-genotoxic non-carcinogens. In response to these requirements, we further evaluated the specificity of the test using two genotoxic non-carcinogens, amaranth (AM) and quercetin dihydrate (QN) in a 4- and/or 28-day treatment regimen, and two non-genotoxic non-carcinogens, NaCl and sucrose (SUC) in a 28-day treatment regimen [15, 20]. However, aneugens and non-genotoxic non-carcinogens have not been evaluated with a 4-day treatment regimen that allows setting higher doses.

To obtain additional data to validate the performance of the GI tract MN test in the present study, we conducted rat glandular stomach, colon, and erythrocyte MN tests using three aneugens—colchicine (COL), vinblastine sulfate (VBS), and docetaxel hydrate (DOC)—and two non-genotoxic non-carcinogens—NaCl (also stomach toxicant) and SUC. These tests were performed with a 4-day treatment regimen using higher doses than those in a long-term treatment study. In the present study with aneugens, MN induction in hepatocytes was preliminarily evaluated.

## Materials and methods

### Animals

Male Crl:CD (SD) rats were purchased from Charles River Laboratories Japan, Inc. (Kanagawa, Japan), acclimated for a week, and used for experiments at an age of 8 weeks. Either two or three rats were housed in a cage with wood-chip bedding, under constant temperature (20 ± 3 °C) and humidity (50 ± 20%) with alternating 12 h intervals of light and dark throughout the acclimation and experimental periods. The rats were provided with food and water ad libitum. All experiments were performed according to the guidelines for the care and use of laboratory animals of the Institutional Animal Care and Use Committee of Yakult Central Institute, and the protocols were approved by the committee.

### Chemicals

We used five test chemicals (COL, VBS, DOC, NaCl and SUC) for evaluation of the performance of the GI tract MN test. The rationale for selecting of these chemicals is as follows; COL, VBS, and DOC were selected as aneugens with different mechanisms of action and routes of administration. COL and VBS exert their action by inhibition of tubulin polymerization [23, 24], while DOC acts mainly through inhibition of tubulin depolymerization [25]. Furthermore, route of administration for COL is oral, while that of VBS and DOC is intravenous injection [24, 26–28]. NaCl and SUC were selected as non-genotoxic non-carcinogens. Additionally, NaCl is a stomach toxicant. Vehicles were used as negative controls and three clastogens, 1,2-dimethylhydrazine dihydrochloride (DMH), *N*-nitroso-*N*-methylurea (MNU), and mitomycin C (MMC), were used as positive controls. Details regarding the test chemicals and positive control chemicals (source, lot number, purity, and vehicle) are given in Table 1. Each chemical was dissolved in its vehicle and administered to rats immediately after preparation.

**Table 1:** Chemical information

Chemicals	Abbreviation	CAS No.	Source	Lot No.	Purity	Vehicle	Dosage (mg/10 mL/ kg body weight/day)	Route
*Aneugens*								
Colchicine	COL	64-86-8	FUJIFILM Wako Pure Chemical	CTP2407	> 95%	DW	4, 8, 16	po
Vinblastine sulfate (Exal® for Inj. 10 mg)	VBS	143-67-9	Nippon Kayaku	Y30400	(PF)	Saline	0.125, 0.25, 0.5	iv
Docetaxel hydrate (TAXOTERE® 20 mg for I.V. Infusion)	DOC	148408-66-6	Sanofi	4D023J	(PF)	Saline	1, 2, 4 ^b)^	iv
*Non-genotoxic non-carcinogens*								
Sodium chloride	NaCl	7647-14-5	FUJIFILM Wako Pure Chemical	KPH4443	≥99.5%	DW	500, 1000, 2000	po
Sucrose	SUC	57-50-1	Tokyo Chemical Industry	E3PLM	> 99%	DW	1000, 5000, 10000	po
*Positive control (clastogens)*								
1,2-Dimethylhydrazine dihydrochloride	DMH	306-37-6	Tokyo Chemical Industry	VU5VG-RD	100%	DW	90	po
*N*-Nitroso-*N*-methylurea	MNU	684-93-5	Sigma-Aldrich	100 M1436	45% ^a)^	DW	20 ^c)^	po
Mitomycin C (MITOMYCIN Injection 2 mg)	MMC	50-07-7	Kyowa Hakko Kirin	569ACH	(PF)	Saline	1	iv

*DW* Water for injection, *PF* Pharmaceutical formulation

^a^The remaining 55% contains stabilizer (water and acetic acid combined)

^b^As amount of docetaxel

^c^As amount of MNU

### Dose levels and treatment

Each treatment group consisted of five randomly selected rats. All administrations were performed in a volume of 10 mL/kg body weight/day. Each animal in the test chemical groups was administered once a day for 4 days (days 1–4), at three doses, via oral gavage (po) or intravenous (iv) consistent with their administration routes for humans (Table 1). The maximum dose of aneugens was determined based on the toxicity data (for example, decrease in body weight) in a preliminary experiment. For non-genotoxic non-carcinogens, the maximum dose was set at 2 g/kg body weight/day (the limit dose in ICH/OECD guidelines [1, 2]) for NaCl and 10 g/kg body weight/day (5-fold of the limit dose) for SUC; these doses correspond to 2/3 and 1/3 of the LD_50_ values [29], respectively. The negative control animals were treated with the vehicle, water for injection (DW, po) or saline (iv), once a day for 4 days. The positive control animals were treated via oral gavage with DMH on day 1 and MNU on days 3 and 4 (P1 regimen). This regimen was demonstrated in our preliminary test to increase the frequency of MNed cells in the liver as well as in the glandular stomach, colon, and bone marrow [15], but not in the peripheral blood. Thus, the positive control animals for the peripheral blood MN test were intravenously treated with MMC for 4 days (P2 regimen).

During the treatment period including the necropsy day, the animals were weighed, and their general condition was observed once a day. On day 3, 24 h after the second administration, approximately 0.1 mL of blood was collected from the tail vein for peripheral blood MN test. Twenty-four hours after the final dosing, the rats were anesthetized with isoflurane and euthanized by exsanguination via abdominal aorta, and their stomachs, colons, livers, and right femurs were obtained.

### Glandular stomach, colon, liver, and bone marrow MN tests

MN tests using the glandular stomach, colon, liver, and bone marrow were performed according to our previous reports of the MMS/JEMS collaborative study [19, 30]. Briefly, the glandular stomach and colon were everted separately on a glass rod and incubated in a solution containing 1 mmol/L ethylenediaminetetraacetic acid disodium salt (EDTA) and 2 mmol/L dithiothreitol for the stomach and 1 mmol/L EDTA for the colon to isolate the epithelium. The liver (left lateral lobe, approximately 1 g) was sliced and incubated in a flask containing 100 units/mL of collagenase (Collagenase Yakult-S; Yakult Pharmaceutical Industry, Tokyo, Japan) to isolate hepatocytes. Bone marrow cells were collected by washing the femur cavity with 1 mL of 10% neutral-buffered formalin. These cell suspensions were washed, fixed with 10% neutral-buffered formalin, and stored at 4 °C until further analysis.

Immediately before observation, the cell suspensions were mixed with staining solution (acridine orange (AO)/4′,6-diamidino-2-phenylindole dihydrochloride mixture for the stomach, colon, and liver; AO solution for the bone marrow) on glass slides. Scoring was carried out under blinded conditions using a fluorescence microscope (magnification: 600× for the stomach, colon, and bone marrow; 400× for the liver) with UV excitation (365 nm) for the stomach, colon, and liver, and with blue excitation (490 nm) for the bone marrow. Two thousand cells or immature erythrocytes (IMEs) were scored per tissue per animal to determine the frequency of MNed cells. Additionally, 1000 erythrocytes from each rat were analyzed to determine the percentage of IMEs among total erythrocytes (%IME).

### Peripheral blood MN test

Peripheral blood MN test was performed using the Rat MicroFlow Plus Micronucleus Analysis Kit (Litron Laboratories, Rochester, MN, USA) and a flow cytometer (BD FACSVerse™ flow cytometer with BD FACSuite™ software, Becton, Dickinson and Company, Franklin Lakes, NJ, USA) following manufacturer’s instructions with slight modification. Briefly, peripheral blood (80 μL) from each animal was mixed with 250 μL anticoagulant/diluent, fixed in ultra-cold methanol, and stored at − 80 °C until further analysis. On the day of analysis, the fixed blood samples were washed and suspended in 20 μL buffer solution. The suspensions (10 μL) were mixed with 40 μL of labeling solution containing RNase solution, rat anti-CD71, and platelet antibody, and kept in the dark for 30 min on ice and then for 30 min at room temperature. The samples were stained with DNA stain solution containing propidium iodide (PI) and immediately used for flow cytometric analysis; flow cytometric analysis acquired approximately 10,000 to 20,000 CD71-positive erythrocytes per animal to determine the frequency of PI/CD71 double-positive erythrocytes (MNed IMEs) and percentage of CD71-positive erythrocytes among total erythrocytes (%IMEs). Before the analysis, a biological standard sample, malaria-infected erythrocytes, was used to set up and calibrate the instrument.

### Ki-67 immunohistochemistry

In the experiment using non-genotoxic non-carcinogens, cell proliferation in the glandular stomach and colon was assessed using Ki-67-positive cells as the marker. We considered that this assay was unsuitable for aneugens known to induce G1, G2, and/or M arrest and, therefore, did not perform the assay.

Ki-67 analysis was performed according to our previous report [20]. Briefly, a part of the glandular stomach (containing the fundus) and colon (middle region, 1 cm) were fixed in 10% neutral-buffered formalin, embedded in paraffin, and cut into 4 μm sections. The sections were deparaffinized and placed in antigen retrieval solution (Target Retrieval Solution; Agilent Technologies Inc., Santa Clara, CA, USA) at 100 °C. Endogenous peroxidase activity was inhibited by incubation with 3% H_2_O_2_. The sections were incubated with monoclonal mouse anti-rat Ki-67 antigen (clone MIB-5; Agilent Technologies Inc.) followed by biotinylated rabbit anti-mouse immunoglobulin (Agilent Technologies Inc.), and subsequently with streptavidin/horseradish peroxidase (Agilent Technologies Inc.). Staining was developed with diaminobenzidine (Agilent Technologies Inc.) and the sections were counterstained with hematoxylin. Scoring was performed using a light microscope (600×).

Thirty glands of the gastric fundus and 30 crypts of the colon were observed to determine the number of Ki-67-positive cells per gland and crypt. A cell was scored positive for Ki-67 when the nucleus of the cell was distinctively brown.

### Statistical analyses

Differences in the MNed cell frequency between the test chemical groups or positive control group and the negative control group were analyzed statistically using Kastenbaum and Bowman’s tables with an upper-tailed significance level of 0.05. When the frequency of MNed cells increased, the Cochran-Armitage test for a dose-related trend was also performed, with a one-sided significance level of 0.05.

The other data were analyzed for statistical significance using two- or multiple-comparison test. Briefly, the statistical significance between two groups was determined using Student’s *t*-test for homogenous data or Aspin-Welch test for non-homogenous data, whereas the statistical significance between multiple groups was determined using Dunnett’s test for homogenous data or Steel test for non-homogenous data, with a two-sided significance level of 0.05. The variance homogeneity of two groups or multiple groups was examined using the *F*-test or Bartlett’s test, respectively, with a two-sided significance level of 0.05.

All analyses were performed using the SAS, version 9.4 (SAS Institute Inc., Cary, NC, USA).

## Results

### Body weight and general condition

**Fig. 1:**
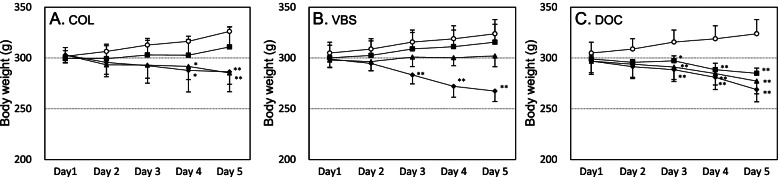
Changes in body weight of different treatment groups: COL (**A**), VBS (**B**), and DOC (**C**). The data are expressed as the mean ± SD. ○: Negative (vehicle) control group, ■: low-dose group (4 mg/kg/day (**A**), 0.125 mg/kg/day (**B**), 1 mg/kg/day (**C**)), ▲: middle-dose group (8 mg/kg/day (**A**), 0.25 mg/kg/day (**B**), 2 mg/kg/day (**C**)), ♦: high-dose group (16 mg/kg/day (**A**), 0.5 mg/kg/day (**B**), 4 mg/kg/day (**C**)). Statistical significance: *p < 0.05, **p < 0.01 (Dunnett’s test) compared to the negative control

A significant decrease in the mean body weight was observed in the treatment groups at middle and high doses of COL, high dose of VBS, and all doses of DOC compared to that of the respective negative control groups (Fig. 1). No change in the general condition was observed in these groups except COL 16 mg/kg body weight/day (hereafter mg/kg) treatment group; in this group, diarrhea was observed in two rats after day 3 and one of them died on the day of necropsy (day 5). In the NaCl and SUC treatment groups, no changes in the mean body weight and general condition were observed compared to the negative control group.

### Glandular stomach, colon, liver, and erythrocyte MN tests

#### Aneugens (COL, VBS, and DOC)

**Fig. 2:**
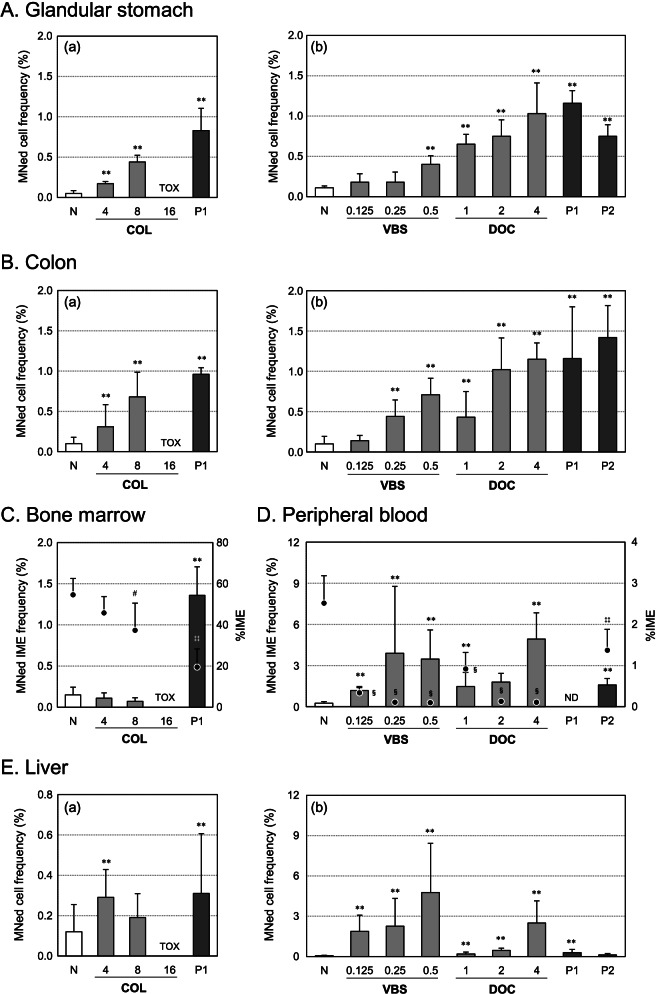
Micronucleus test in the glandular stomach, colon, bone marrow, peripheral blood, and liver with aneugens. The test was performed in rats administered COL (po), VBS (iv), and DOC (iv) for 4 days. The sampling points were as follows: 24 h after 4 daily administration (stomach, colon, bone marrow, and liver), and 24 h after the second administration (peripheral blood). Each bar represents the frequency of MNed cells or MNed immature erythrocytes (IMEs) (mean ± SD). Each closed circle represents %IME (mean ± SD). The horizontal axis represents chemical and/or dosage (mg/kg/day). N, Negative (vehicle) control; P, positive control (P1: MNU and DMH treatment, P2: MMC treatment); TOX, data were excluded due to severe toxicity; ND, not done. Statistical significance: **p < 0.01 (Kastenbaum & Bowman test), ^#^*p* < 0.05 (Dunnett’s test), ^§^*p* < 0.05 (Steel test), and ^‡‡^*p* < 0.01 (Aspin-Welch test) compared to the negative control

Two animals administered 16 mg/kg COL exhibited severe toxicity (death and/or diarrhea); therefore, we judged this dosage as inappropriate for genotoxicity evaluation and used the samples from animals administered 4 and 8 mg/kg COL for MN analysis. In the glandular stomach and colon of the animals administered COL orally, statistically significant and dose-dependent increases in the MNed cell frequencies were observed at doses of 4 and 8 mg/kg compared to those of the respective negative controls (Fig. 2A(a) and B(a)). In the bone marrow, no significant increase in the MNed IME frequency was observed at any dose; however, %IME decreased significantly at 8 mg/kg, providing evidence for the bone marrow exposure (Fig. 2C). In the liver, although the frequency of MNed cells increased in both the 4 and 8 mg/kg groups, a statistically significant increase was observed only in the 4 mg/kg group; this increase in frequency for both groups was not dose-dependent (Fig. 2E(a)).

In the animals administered VBS and DOC intravenously for 4 days, statistically significant and dose-dependent increases in the MNed cell frequencies were observed in the glandular stomach and colon (Fig. 2A(b) and B(b)). In the bone marrow, cell proliferation was markedly inhibited (% IME values were less than 10% of the negative control values) at all dosages after 4-day treatment; hence, the MNed cell frequencies could not be analyzed. Instead, MN induction was analyzed using peripheral blood collected on the day after the second treatment and a statistically significant and dose-dependent increase in the frequency of MNed IMEs was observed (Fig. 2D). The %IMEs decreased significantly at all doses for each chemical. In the liver, statistically significant and dose-dependent increases in MNed cell frequencies were observed at all doses of both VBS and DOC after 4-day treatment.

#### Non-genotoxic non-carcinogens (NaCl and SUC)

**Fig. 3:**
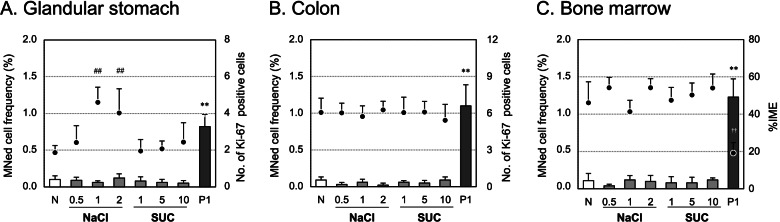
Micronucleus test in the glandular stomach, colon, and bone marrow with non-genotoxic non-carcinogens. The test was performed in rats administered NaCl or SUC for 4 days. Each bar represents the frequency of MNed cells or MNed immature erythrocytes (IMEs) (mean ± SD). Each closed circle represents the number of Ki-67 positive cells (per gland or crypt) or %IME (mean ± SD). The horizontal axis represents chemical and/or dosage (mg/kg/day). N, Negative (vehicle) control; P1, positive control (DMH and MNU treatment). Statistical significance: **p < 0.01 (Kastenbaum & Bowman test), ^##^*p* < 0.01 (Dunnett’s test), and ^††^*p* < 0.01 (Student’s *t*-test) compared to the negative control

In the NaCl and SUC treatment groups, no significant increase in MNed cell frequency was observed in any of the three tissues (Fig. 3). In the NaCl treatment group, the number of Ki-67-positive cells significantly increased only in the glandular stomach at doses of 1 and 2 mg/kg, while in the SUC treatment group, no change in cell proliferation was observed in any of the three tissues.

#### Positive control

In all of the P1 positive control groups (DMH + MNU treatment groups) set for the glandular stomach, colon, bone marrow, and liver MN tests, statistically significant increases in MNed cell frequencies were observed in all target tissues (Figs. 2, 3). In addition, in the P2 positive control group (MMC treatment group) set for the peripheral blood MN test, statistically significant increase in the MNed cell frequencies were observed not only in the peripheral blood but also in the glandular stomach and colon (Fig. 2).

## Discussion

We assessed the performance of the GI tract MN test for aneugens (COL, VBS, and DOC) and non-genotoxic non-carcinogens (NaCl and SUC) with a 4-day treatment regimen. The results of MN tests, in vitro genotoxicity tests, and carcinogenicity studies are shown in Table 2 on several chemicals that were used in this study and past studies [15, 18, 30–51].

**Table 2:** Summary of the results of 4-day repeated-dose micronucleus test

Classes	Chemicals	Route	Micronucleus test with a 4-day treatment regimen				In vitro genotoxicity test		Carcinogenicity in rodent (main target) [Ref.]
			Glandular stomach	Colon	Bone marrow	Ref.	Ames [Ref.]	Chromosomal aberration [Ref.]	
Aneugens	COL	po	+	+	–	Present study	– [31]	+ (poly) [31]	No data
	VBS	iv	+	+	Tox (PB; +) ^a)^	Present study	– [31]	+ (poly) [31, 32]	No data
	DOC	iv	+	+	Tox (PB; +) ^a)^	Present study	– [33]	+ (poly) [33]	No data
Clastogens	MNU	po	+	+	+	[15]	+ [34]	+ [34]	+ (Stomach) [35, 36]
	4NQO	po	+	–	+	[15]	+ [31]	+ [31]	+ (Stomach) [37]
	MNNG	po	+	–	–	[15]	+ [34]	+ [34]	+ (Stomach) [35, 38]
	NMUT	po	+	–	–	[15]	+ [39]	+ [39]	+ (Stomach) [18]
	DMH	po	–	+	+	[15]	+ [34]	+ [34]	+ (Colon) [35, 40]
	PhIP	po	–	+	+	[15]	+ [31]	+ [31]	+ (Colon) [35, 41]
	KBrO_3_	po	+	Eq	+	[15, 30]	+ [42]	+ [42]	+ (Kidney) [35, 42]
	MMC	iv	+	+	+	Present study	+ [31, 34]	+ [31, 34]	+ (Peritoneum) [35]
Genotoxic non-carcinogens	AM	po	–	–	–	[15]	– [43]	+ [39]	– [44, 45]
	QN	po	–	–	–	[15]	+ [43]	+ [43]	– [46–48]
Non-genotoxic non-carcinogens	NaCl	po	–	–	–	Present study	– [34]	– [34], + [49]	– [34, 35]
	SUC	po	–	–	–	Present study	– [50]	– [51], + [49]	– [35]

+, Positive; −, negative; *Eq* Equivocal, *Tox* Toxic, *PB* Peripheral blood, *Ref.* References

^a^MN induction in erythrocytes was analyzed using peripheral blood on the day after the second administration

*COL* Colchicine, *VBS* Vinblastine sulfate, *DOC* Docetaxel hydrate, *MNU N*-nitroso-*N*-methylurea, *4NQO* 4-nitroquinoline-1-oxide, *MNNG N*-methyl-*N′*-nitro-*N*-nitrosoguanidine, *NMUT N*-methyl-*N*-nitrosourethane, *DMH* 1,2-dimethylhydrazine dihydrochloride, *PhIP* 2-amino-1-methyl-6-phenylimidazo[4,5-*b*]pyridine hydrochloride, *KBrO*_*3*_ Potassium bromate, *MMC* Mitomycin C, *AM* Amaranth, *QN* Quercetin dihydrate, *NaCl* Sodium chloride, *SUC* Sucrose

COL, an alkaloid product of *Colchicum autumnale*, inhibits tubulin polymerization, and it is used as an oral medicine to treat gout and other inflammations [26, 27]. VBS, a vinca alkaloid, and DOC, a taxoid, are known to inhibit tubulin polymerization and depolymerization, respectively, and are used as anti-cancer drugs administered intravenously [24, 25, 28]. After administering these aneugens to rats according to their clinical administration route, the frequencies of MNed cells in the glandular stomach and colon significantly increased in a dose-dependent manner for all aneugens. It is considered that intravenously administered VBS and DOC reach the stomach and colon via the bloodstream and then induce MN in the cells of these organs. Being administered intragastrically, COL was in contact with the stomach. After absorption from the small intestine, a proportion of the unchanged drug possibly reaches the stomach and colon via the blood stream, and the major proportion reaches the colon from the luminal side due to the involvement of P-glycoprotein in the small intestine and liver [27]. In the present study, it was revealed that the GI tract MN test could detect aneugens in the glandular stomach and colon of rats when administered via clinical route (intravenous and oral administration). To date, there are few reports of MN induction by aneugens in GI tract cells, particularly in the stomach epithelium. Therefore, the present results provide important evidence showing good sensitivity of the GI tract MN test to aneugens.

In erythrocytes, on the other hand, MN induction could not be detected even when COL was administered up to the maximum tolerated dose. Cammerer et al. [52] reported a delayed increase in MNed erythrocytes (peak at 96 h after the final dosing) in rats orally administered 6 and 8 mg/kg COL for five consecutive days. In addition, in the present study, since severe cytotoxicity was observed in the bone marrow of rats administered VBS or DOC for 4 days at all dosages, MNed erythrocyte induction could not be assessed in the bone marrow (data not shown). Instead, MN induction was detected in the peripheral blood on the day after the second administration. Therefore, the 4-day treatment regimen allows to collect peripheral blood during the dosing period or collect bone marrow cells after the recovery period to evaluate MNed erythrocyte induction by compounds such as aneugens that cause severe bone marrow toxicity and/or delay of MN induction.

The MNed hepatocyte frequencies in rats treated with aneugens were preliminarily monitored in the present study, and dose-dependent MN induction (positive response) was confirmed for VBS and DOC. For COL, although the frequency of MNed cells increased in both the 4 and 8 mg/kg groups, a statistically significant increase was observed only in the 4 mg/kg group; this increase in frequency for both groups was not dose-dependent. The MN test using the liver, the primary tissue involved in metabolism, as well as the GI tract MN test are considered important for in vivo genotoxicity evaluation. Therefore, various methods such as the partial hepatectomy (PH) method, the juvenile/young rat method, and the RD method have been developed [22, 53, 54]. Sensitivity verification using clastogens has been carried out using these methods but that using aneugens has been mainly performed using the PH method. Currently, the MMS group is evaluating the sensitivity of the liver MN test with the 28-day RD method using aneugens (Shigano M et al., manuscript in preparation). The RD method has the potential to detect MN induction in cells in several tissues including the liver, bone marrow, and GI tract. However, the cell division cycles differ substantially among these tissues, and in the case of aneugens that induce mitotic inhibition and severe cytotoxicity, it is important to determine the optimal dosage, dosing period, and sampling time for MN analyses in the respective tissues. In the present study, a significant increase in MNed cell frequency was observed in the liver in addition to the GI tract and peripheral blood in rats administered aneugens with a 4-day treatment regimen. These results suggest that it may be possible to detect the aneugenic potential of chemicals in the liver using this 4-day treatment regimen. However, further research is required to elucidate the performance of this short-term administration method.

To investigate the specificity of the GI tract MN test with a 4-day treatment regimen, NaCl and SUC were selected as non-genotoxic non-carcinogens. They did not induce MNed cells in the GI tract and bone marrow of rats when administered up to 2 g/kg of NaCl (2/3 of the LD_50_ value) and 10 g/kg of SUC (1/3 of the LD_50_ value) [29]. No treatment-related changes were observed in the clinical signs or body weight gain. NaCl is a stomach toxicant that promotes the proliferation of the glandular stomach epithelium [55] and exerts tumor-promoting activity [56] when orally administered to rats. SUC is known to enhance the proliferation of the colonic epithelium of rats administered orally [57]. As an index of cell proliferation in the GI tract, we analyzed proliferating cells in tissue sections using immunohistochemistry of Ki-67, a nuclear protein expressed in the cell cycle phase other than the G0 phase [58]. In the present study, the Ki-67-positive cell counts significantly increased only in the gastric epithelium of the rats treated with NaCl, indicating that cell proliferation was enhanced, but not in the cells of all three tissues evaluated including the colon in the rats treated with SUC. It has been reported that an increase in erythropoiesis that is not due to chemical-induced genotoxic damage causes an increase in the number of spontaneous MNed erythrocytes, resulting in false-positive results in the routine erythrocyte MN test [59]. Under the conditions of the present experiment, however, it was demonstrated that both NaCl and SUC did not increase MNed cell frequencies in the glandular stomach and colon, regardless of whether cell proliferation was enhanced. These results show that non-genotoxic non-carcinogens including chemicals that enhance cell proliferation could be correctly evaluated as negative in the glandular stomach and colon MN tests.

In our previous study, the rat glandular stomach and colon MN tests with a 4-day treatment regimen were performed using seven clastogens and two genotoxic-non-carcinogens, and their sensitivity and specificity were confirmed (Table 2). Furthermore, the present study revealed that aneugens can be detected as positive, and non-genotoxic non-carcinogens can be evaluated as negative. Based on these results, the glandular stomach and colon MN tests with a 4-day regimen showed to have good sensitivity and specificity in the evaluation of genotoxicity (clastogenicity and aneugenicity) of test chemicals.

## Conclusions

To further clarify the sensitivity and specificity of the rat GI tract MN test, we conducted additional studies using three aneugens and two non-genotoxic non-carcinogens with a 4-day treatment regimen. The study demonstrated that the glandular stomach and colon MN tests could detect aneugens as positive and could adequately evaluate non-genotoxic non-carcinogens as negative, including a chemical that enhances cell proliferation. These results provide important evidence supporting the good performance of the rat glandular stomach and colon MN tests with a 4-day treatment regimen.

## Data Availability

All data generated or analyzed during this study are included in this published article.

## Abbreviations

MN: Micronucleus
GI: Gastrointestinal
RD: Repeated-dose
COL: Colchicine
VBS: Vinblastine sulfate
DOC: Docetaxel hydrate
SUC: Sucrose
DMH: 1,2-dimethylhydrazine dihydrochloride
MNU: *N*-nitroso-*N*-methylurea
MMC: Mitomycin C
